# Fluorescence intensity correlation imaging with high spatial resolution and elemental contrast using intense x-ray pulses

**DOI:** 10.1063/4.0000105

**Published:** 2021-07-29

**Authors:** Phay J. Ho, Christopher Knight, Linda Young

**Affiliations:** 1 Chemical Sciences and Engineering Division, Argonne National Laboratory, Lemont, Illinois 60439, USA; 2 Computational Science Division, Argonne National Laboratory, Lemont, Illinois 60439, USA; 3 Department of Physics and James Franck Institute, The University of Chicago, Chicago, Illinois 60637, USA

## Abstract

We theoretically investigate the fluorescence intensity correlation (FIC) of Ar clusters and Mo-doped iron oxide nanoparticles subjected to intense, femtosecond, and sub-femtosecond x-ray free-electron laser pulses for high-resolution and elemental contrast imaging. We present the FIC of Kα and $K\alpha^{h}$ emission in Ar clusters and discuss the impact of sample damage on retrieving high-resolution structural information and compare the obtained structural information with those from the coherent diffractive imaging (CDI) approach. We found that, while sub-femtosecond pulses will substantially benefit the CDI approach, few-femtosecond pulses may be sufficient for achieving high-resolution information with the FIC. Furthermore, we show that the fluorescence intensity correlation computed from the fluorescence of the Mo atoms in Mo-doped iron oxide nanoparticles can be used to image dopant distributions in the nonresonant regime.

## I. INTRODUCTION

Coherent diffractive imaging (CDI)^1,2^ with x-ray free-electron laser (XFEL) pulses holds the promise to probe the structure^3,4^ and follow the dynamics^5–7^ of entities with atomic resolution.^1^ This approach is based on the “probe before destroy” concept and makes use of the very high number of photons in a single pulse. The idea is that with short-duration pulses, high-resolution diffraction patterns can be captured with a single XFEL pulse before the system of interest suffers damage from the intense radiation.^1^ Despite continuous progress, it has remained a challenge^8,9^ to achieve nanometer or subnanometer resolution and elemental contrast^10^ with CDI. This is because intense x-ray pulses will lead to extremely rapid structural degradation of the sample and generate a large number of delocalized electrons, resulting in a substantial reduction in both scattering efficiency^9,11^ and signal-to-noise in the measured scattering patterns.^12–14^

To circumvent the challenges of the CDI approach, new XFEL imaging modalities are being developed for single-particle imaging (SPI). Recently, an approach using single-shot fluorescence intensity correlation (FIC) has been proposed to measure the 3D distribution of heavy elements using intense x-ray pulses.^15^ This approach was inspired by the Hanbury Brown and Twiss (HBT) effect,^16^ which has enabled the determination of the size of astronomical objects from the intensity correlation of their emitted light. The discovery of the HBT effect has pushed the imaging resolution below the Abbe limit (classical diffraction limit) in the visible range,^17–23^ as well as in the Extreme ultraviolet (XUV) regime.^24^ Also, recent experiments demonstrated that the FIC can be used to directly determine the structure of trapped ion pairs.^25,26^ We point out that an alternative fluorescence imaging approach has been proposed by Ma and co-workers to measure the molecular structure from the interference of the emitted fluorescence from x-ray excited molecules with identical atoms.^27,28^ Unlike FIC, this approach is related to measuring the first-order field correlation functions.

The HBT effect is a two-photon interference phenomenon resulting from two indistinguishable pathways for photons emitted from different source points reaching two different detectors.^16^ Such indistinguishability occurs if the two photons are emitted within the coherence time of the emitted light. In the case that the emission time window is longer than the coherence times, the visibility of the interference fringes is reduced.^15,29^ In principle, a pair of atoms excited by an XFEL pulse with a pulse duration shorter than the coherence time of the fluorescence states can lead to the indistinguishable condition; in that case, both atoms relax via fluorescence and the time interval between the two photons will be within the coherence time. For atomic-scale imaging, the FIC approach works best with monochromatic fluorescence events. We point out that while pixelated energy resolving detectors are not in current use at XFEL or synchrotron facilities, such detectors are currently under development for the x-ray astronomy community.^30^

With the ever-improving XFEL and detector technology and the emerging new operation modes that deliver intense few- and sub-femtosecond pulses at high repetition rates,^31^ the XFEL will further accommodate this photon-hungry fluorescence intensity correlation measurement^24^ in both the soft and hard x-ray regimes. In the past few years, several research groups have begun experimental and theoretical studies of fluorescence imaging that employs XFEL pulses. For example, the FIC method has been used to determine both the pulse duration^32^ and focal area^33^ of femtosecond XFEL pulses using Kα fluorescence of copper while filtering out K_*β*_. Recent simulation work by Trost and co-workers suggests that while it is challenging to image macroscopic objects due to low signal-to-noise ratios, the FIC may be useful for imaging of single particles.^34^ Our previous study, which used Ar clusters as a prototypical system, showed that the intense, few-femtosecond XFEL pulses can enable a multitude of atomic fluorescence channels, but their dynamics in an extended system are drastically different from that in isolated atoms. We found that the x-ray emission time in extended systems is dictated by not only the x-ray pulse duration and a lifetime of the core-excited states—rather—ultrafast electron dynamics can greatly modify fluorescence characteristics by opening additional fluorescence pathways via electron–ion recombination in addition to the direct photoionization pathways. We point out that the recombination pathways have been observed in Ar clusters in the XUV regime.^35^ The existence of these two pathways gives rise to higher Kα and $K\alpha^{h}$ yields and broader temporal emission profiles in clusters. Here, Kα is the fluorescence from a singly charged ion with a K-shell vacancy and a 2p electron fills the K-shell vacancy, whereas $K\alpha^{h}$ is the fluorescence from a doubly charged ion with a hollow K shell and a 2p electron fills a K-shell vacancy. Our calculation suggested that $K\alpha^{h}$ can be advantageous for FIC imaging relative to Kα, since $K\alpha^{h}$ emission takes place before the onset of significant structural damage.

Building on the results of our previous work, we theoretically explore FICs for high-resolution and elemental contrast imaging of isolated nanosized systems with intense x-ray pulses. We investigate the FIC of Kα and $K\alpha^{h}$ emission in Ar clusters as a function of pulse duration and discuss the impact of sample damage on the FIC of these two emission lines and the computed FICs. Also, we show that the FIC is less sensitive to radiation damage than CDI and can be exploited for retrieving high-resolution structural information. Furthermore, we examined the FICs computed from the fluorescence of the Mo atoms in Mo-doped iron oxide hollow core-shell nanoparticles (NPs). In these particles, the macroscopic properties and catalytic functions are sensitive to the presence of Mo dopants and the structure of the NPs.^36^ However, the influence of the dopant is not yet known as the atomic-scale structures and, in particular, the dopant distributions in the shell are not known.^36,37^ Our calculations show that the FIC is more sensitive to the dopant distribution than CDI in the non-resonant regime.

## II. MODEL

To model the FIC of an extended system, we treat individual fluorescent atoms as random light emitters, such that the intensity of the combined emitted radiation field at $\vec{k}$ can be expressed as

$$I(\vec{k})=I_{0}\sum_{j=1}^{N_{\mathrm{emitters}}}\sum_{l=1}^{N_{\mathrm{emitters}}}\beta_{j}\beta_{l}^{*}e^{i\vec{k}\cdot (\vec{r}-{\vec{R}}_{f,j})}e^{-i\vec{k}\cdot (\vec{r}-{\vec{R}}_{f,l})}e^{i(\phi_{j}-\phi_{l})},$$ (1)

where ${\vec{R}}_{f,j}$ is the position and $\phi_{j}$ the random phase of the *j*th emitter. Here, $\phi_{j}$ is related to the emission time of the random fluorescence event. $I_{0}=\langle I(\vec{k},\{{\vec{R}}_{f,j}\})\rangle$, which is independent of $\vec{k}$, is the average intensity obtained by averaging over many realizations of the random fluorescence events, and *β_j_* is considered as the fractional emitter strength such that

$$\sum_{j}^{N_{\mathrm{emitter}}}|\beta_{j}{|}^{2}=1.$$ (2)

For a collection of noninteracting emitters (i.e., in the gas phase), $|\beta_{j}{|}^{2}=1/N_{\mathrm{emitter}}$. Yet, for an extended and condensed system in an intense x-ray pulse, this is not true and *β_j_* can depend on the location of the emitter. This is because the x ray-induced ionization and the subsequent electron–ion recombination and massive electron rearrangement can quickly transform the exposed sample volume into a nonuniform spatial charge density structure (neutral core and highly charged shell).^38,39^

In this model, the FIC is the second-order correlation function of the fluorescence intensity measured at two momentum transfer vectors, $I({\vec{k}}_{1}$) and $I({\vec{k}}_{2})$, and is related to the Fourier transform of the distribution of the fluorescence emitters *ρ_f_* by the following relation:^29,40^

$$G_{2}({\vec{k}}_{1},{\vec{k}}_{2},\{{\vec{R}}_{f,j}\})-1=|\int d^{3}r\rho_{f}(\vec{r},\{{\vec{R}}_{f,j}\})e^{i{\vec{q}}_{f}\cdot \vec{r}}{|}^{2},$$ (3)

where ${\vec{q}}_{f}={\vec{k}}_{1}-{\vec{k}}_{2}$ is the momentum transfer vector, and $\left\{{\vec{R}}_{f,j}\right\}$ denotes the spatial configuration of all the emitters and

$$G_{2}({\vec{k}}_{1},{\vec{k}}_{2},\{{\vec{R}}_{f,j}\})=\frac{\left\langle I({\vec{k}}_{1},\{{\vec{R}}_{f,j}\})I({\vec{k}}_{2},\{{\vec{R}}_{f,j}\})\right\rangle }{\left\langle I({\vec{k}}_{1},\{{\vec{R}}_{f,j}\})\rangle \langle I({\vec{k}}_{2}\{{\vec{R}}_{f,j}\})\right\rangle },$$ (4)

$$\rho_{f}(\vec{r},\{{\vec{R}}_{f,j}\})=\sum_{j}^{N_{\mathrm{emitter}}}|\beta_{j}{|}^{2}\delta (\vec{r}-{\vec{R}}_{f,j}).$$ (5)

However, we note that Eqs. (3) and (4) do not account for the temporal coherence of Kα and $K\alpha^{h}$ radiation. If the duration of the temporal emission profile, *τ_F_*, is longer than the coherence time, *τ_c_*, of the fluorescence, which is given by the core-hole lifetime, the visibility of the FIC will be reduced roughly by $\tau_{F}/\tau_{C}$.^29,34^

An intense x-ray pulse can strongly excite the sample leading to the changes in the spatial arrangement of the emitters during the time window of fluorescence. The measured *G*_2_ needs to be weighted over all the spatial configurations

$${\left\langle G_{2}({\vec{k}}_{1},{\vec{k}}_{2},\{{\vec{R}}_{f,j}\})\right\rangle }_{\mathrm{avg}}=\sum_{\left\{{\vec{R}}_{f,j}\right\}}p(\{{\vec{R}}_{j}\})G_{2}({\vec{k}}_{1},{\vec{k}}_{2},\{{\vec{R}}_{f,j}\}),$$ (6)

where $p(\{{\vec{R}}_{f,j}\})$ is the probability of the collection of emitters in the configuration of $\left\{{\vec{R}}_{f,j}\right\}$ and $\sum_{\left\{{\vec{R}}_{f,j}\right\}}p(\{{\vec{R}}_{f,j}\})=1$.

To determine *G*_2_ in Eq. (6), we used our previously developed Monte Carlo/molecular dynamics (MC/MD) method. This is an effective method for describing matter interacting with intense x-ray pulses, and it has been used to reproduce XFEL experimental data: ion kinetic energy distribution of Ar clusters,^39^ ion charge state distribution of Ar atoms,^41^ and the ultrafast x-ray scattering response of molecular clusters.^9^ The details of this method are described in our previous work.^11,39^ In brief, the interaction of the cluster of atoms with the incident XFEL pulse is treated quantum mechanically with a Monte Carlo method by tracking explicitly the time-dependent quantum transition probability between different electronic configurations. The total transition rate, Γ, between different electronic configurations *I* and *J* is given by

$$\Gamma_{I,J}=\Gamma_{I,J}^{P}+\Gamma_{I,J}^{A}+\Gamma_{I,J}^{F}+\Gamma_{I,J}^{RE}+\Gamma_{I,J}^{EI}+\Gamma_{I,J}^{RC}.$$ (7)

Starting from the ground state of the neutral atom, we include the contribution from photoionization $\Gamma_{I,J}^{P}$, Auger decay $\Gamma_{I,J}^{A}$, fluorescence $\Gamma_{I,J}^{F}$, resonant excitation $\Gamma_{I,J}^{RE}$, electron-impact ionization $\Gamma_{I,J}^{EI}$, and electron–ion recombination $\Gamma_{I,J}^{RC}$. In addition, a molecular dynamics (MD) algorithm is used to propagate all particle trajectories (atoms/ions/electrons) forward in time. Our model assumes the K-shell fluorescence emission is isotropic. By tracking the time evolution of electronic configurations of atoms and ions (timing and energy of each fluorescence event) and their positions, MC/MD can study the impact of an intense x-ray pulse on dynamics and temporal emission profile of various fluorescence channels, as well as the associated FICs, in one calculation.

**FIG. 1.**
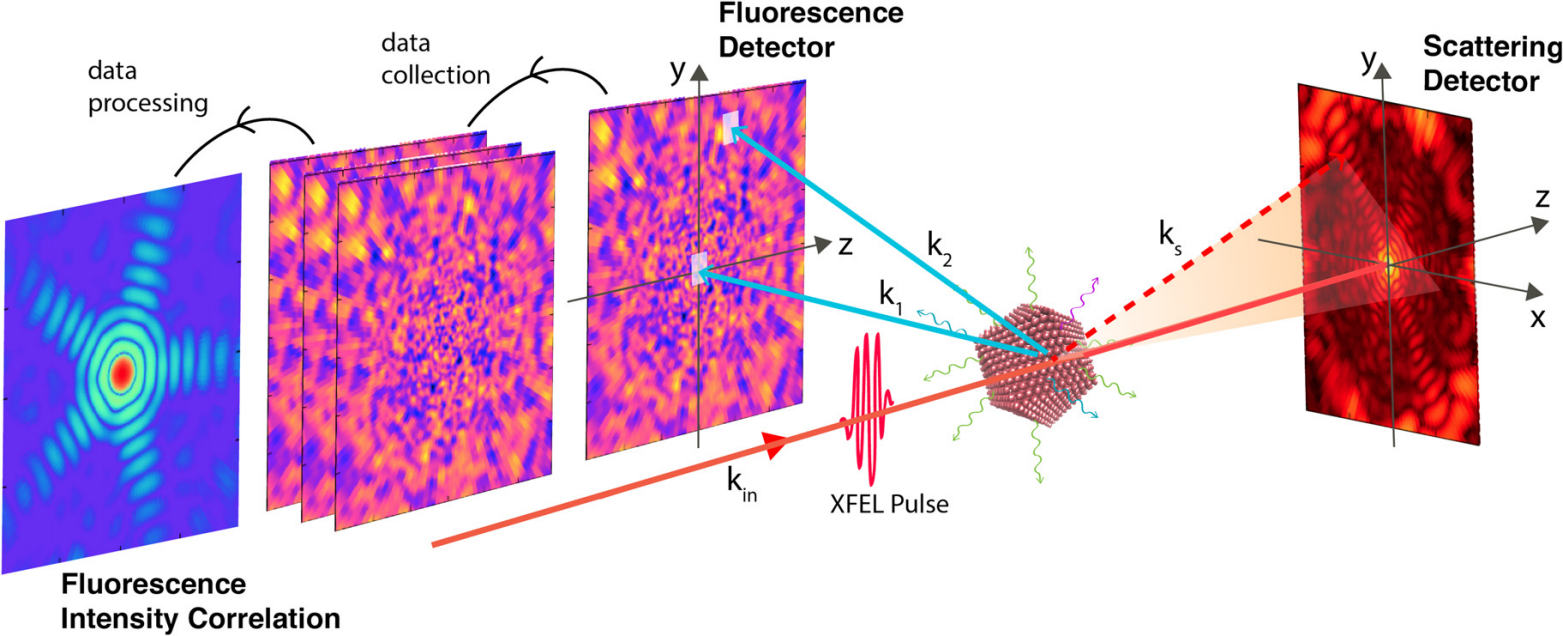
Setup of SPI with coincident fluorescence and scattering imaging. The fluorescence detector is placed perpendicular to the x-ray beam, where the coherent scattering is suppressed. In principle, it can be placed independent of the beam since fluorescence emission is isotropic. In FIC, the structural information is obtained from the correlation of many pairs of spatially separated pixels.

In this paper, we further exploit the capability of the MC/MD model to investigate multiple imaging modalities. In addition to the FIC, we also examine the x-ray scattering pattern under the same pulse conditions. The scattering response is modeled as a sum of the instantaneous scattering patterns weighted by the pulse intensity, $j_{X}(\tau ,t)$ with FWHM duration *τ*. In our model, the scattering signals expressed in terms of the total differential scattering cross section of the target system can be regarded as the sum of the coherent (elastic) and incoherent (inelastic) scattering^42–45^

$$\frac{d\sigma_{\mathrm{total}}}{d\Omega }(\vec{q})=\frac{d\sigma_{\mathrm{coh}}}{d\Omega }(\vec{q})+\frac{d\sigma_{\mathrm{incoh}}}{d\Omega }(\vec{q}),$$ (8)

where the coherent scattering can be expressed as

$$\frac{d\sigma_{\mathrm{coh}}}{d\Omega }(\vec{q})=\frac{d\sigma_{th}}{d\Omega }\frac{1}{F}\int_{-\infty }^{+\infty }dtj_{X}(\tau ,t)|F_{c}(\vec{q},t){|}^{2},$$ (9)

where $d\sigma_{th}/d\Omega$ is the Thomson scattering cross section and $F=\int_{-\infty }^{+\infty }dtj_{X}(\tau ,t)$ is the fluence of an XFEL pulse. Here,

$$F_{c}(\vec{q},t)=\int d^{3}\vec{r}\rho_{1e}(\vec{r};\{{\vec{R}}_{j}\},t)e^{i\vec{q}\cdot \vec{r}}$$ (10)

is the time-dependent form factor of the target system, where $\rho_{1e}(\vec{r};\{{\vec{R}}_{j}\},t)$ is the time-dependent electron density of the system with a geometry, $\left\{{\vec{R}}_{j}\right\}$. In this reference frame, as shown in Fig. 1, the momentum transfer vector is $\vec{q}={\vec{k}}_{in}-{\vec{k}}_{s}$, where ${\vec{k}}_{in}$ and ${\vec{k}}_{s}$ are the wave vectors of the incident and scattered photons.

By using the independent atom model (IAM), $F_{c}(\vec{q},t)$ can be written as

$$F_{c}(\vec{q},t)=\sum_{j=1}^{N_{a}}f_{j}(\vec{q},C_{j}(t))e^{i\vec{q}\cdot {\vec{R}}_{j}(t)}+\sum_{j=1}^{N_{e}(t)}e^{i\vec{q}\cdot {\vec{r}}_{j}(t)},$$ (11)

where *N_a_* is the total number of atoms/ions, ${\vec{R}}_{j}(t),\;C_{j}(t)$, and $f_{j}[\vec{q},C_{j}(t)]$ are the position, the electronic configuration, and the atomic form factor of the *j*th atom/ion, respectively. $N_{e}(t)$ is the number of delocalized electrons within the focal region of the x-ray pulse, and ${\vec{r}}_{j}(t)$ are their positions. Our previous work shows that the IAM works well for describing the intense-pulse scattering response of molecular clusters^9^ and single molecules, in which the difference between the IAM and density functional theory (DFT) methods, which go beyond IAM and include electron correlations, is of the order of a few percent.^46^

The contribution from the incoherent scattering processes is cast in terms of the incoherent scattering function, $S(\vec{q},t)$^42,45^

$$\frac{d\sigma_{\mathrm{incoh}}}{d\Omega }(\vec{q})=\frac{d\sigma_{th}}{d\Omega }\frac{1}{F}\int_{-\infty }^{+\infty }dtj_{X}(\tau ,t)S(\vec{q},t),$$ (12)

with

$$S(\vec{q},t)=\sum_{j=1}^{N_{a}}s_{j}(\vec{q},C_{j}(t)),$$ (13)

and $s_{j}[\vec{q},C_{j}(t)]$ is the incoherent scattering function of the *j*th atom/ion with electronic configuration $C_{j}(t)$. Our method can also include the effect of the bandwidth of the XFEL pulse^9^ on the scattering response by convolving the differential cross section with the bandwidth profile.

Using the MC/MD method, we examine FIC and CDI of Ar_1415_ and Ar_149171_ clusters in an intense 5 keV pulse with a fluence of 3.5 × 10^12^ photons/*μ*m^2^, which corresponds to 10 times the fluence for saturating single ionization of Ar. Both Ar_1415_ and Ar_149171_ are constructed as icosahedral structures with 7 and 35 geometric shells.^47^ These clusters are initially oriented with a fivefold symmetric axis along the x axis (z axis is the x-ray propagation axis). The diameters of Ar_1415_ and Ar_149171_ are 5.3 and 26.3 nm. The choice of pulse fluence and photon energy is motivated by our previous work with a 2 fs pulse, in which these parameters can enable high Kα and $K\alpha^{h}$ yield in a femtosecond temporal emission window.^48^ Motivated by the capability to generate pulses with different pulse lengths, we examine the pulse duration dependence of FIC and CDI in the range of 0.25–10 fs.

Our goal is to explore the feasibility of controlling the Kα and $K\alpha^{h}$ temporal emission profile and contrast of the interference fringes with intense XFEL pulse, using pulse duration as a control knob. We are interested in a regime, where the ionization rate of a single core-hole state (SCHS) is comparable or higher than the inner-shell decay rate. In this regime, the width of the temporal emission profile (at least for Kα) in isolated atoms will shrink with decreasing pulse duration (increasing pulse intensity) due to the increasing depletion rate of the single core-hole state. For extended systems, the pulse duration dependence of the fluorescence emission is not trivial due to the complex x-ray excitation dynamics in these systems. For this study, our targets are Ar clusters. To explore the feasibility of imaging elemental contrast with the FIC, we investigate the scattering and fluorescence response for imaging dopant distribution in γ− Fe_2_O_3_ with intense x-ray pulses.

In this work, our photon energy is 5 keV, and within the pulse spectral width, we do not access any resonances in Ar clusters and the doped iron oxide nanoparticles. Thus, here the spectral width does not have a significant effect on the resulting fluorescence and scattering response. In our pulse duration dependence study, we point out that required pulse intensities can be reduced by about a factor of 5 if a lower photon energy is used [close to the K-edge of a double core-hole state (DCHS) of Ar^2+^] to achieve 10 times the single ionization saturation fluence. In such a near-resonance case, the effect of spectral width will be important and it will induce additional complexities in the pulse duration dependence study.

Due to the small probability of fluorescence and the Monte Carlo nature of the calculation, a large number of replicas are needed to produce statistically converged fluorescence data. For our calculations, we used 100 to 10 000 replicas such that the error in per atom fluorescence yield of the fluorescence channels of interest is less than 0.1%. In particular, for the Ar_1415_ and Ar_149171_ clusters, we used 10 000 and 100 replicas, respectively. For all the iron oxide nanoparticle calculations, 1000 replicas were used. A time step of 2 as is found to be sufficient to follow the electron processes and nuclear dynamics.^48^ For each calculation, we propagate the system dynamics for tens of femtoseconds as the fluorescence temporal emission extends beyond the pulse duration.

We note that from a given 2D fluorescence intensity detection from an excited target in a fixed orientation, as shown in Fig. 1, a large 3D FIC data set can be gathered from all accessible pairs of ${\vec{k}}_{1}$ and ${\vec{k}}_{2}$.^15^ This means one can gather the full 3D data set needed for 3D reconstruction by measuring fluorescence from a small number of sample orientations. To illustrate the impact of intense-field XFEL on the FIC, we focus on a subset of FIC data, in which ${\vec{k}}_{1}$ is fixed along the −x axis and ${\vec{k}}_{2}$ scans across all detector pixels on the y-z plane, as shown in Fig. 1. The fluorescence detector is placed perpendicular to the XFEL propagation, where the probability of coherent scattering is suppressed.

Using a coincident FIC and CDI setup, one might be able to deduce the sample size, target orientation and at the same time measure the atomic structure, paving ways to achieve high-fidelity 3D structure reconstruction. For example, FIC can serve to inform the relative sample orientation, whereas 2D scattering patterns can determine the size of the exposed particles.^6,9,49^

## III. RESULTS

### A. Ar clusters

We begin our discussion by showing the fluorescence intensity and the FIC associated with the Kα and $K\alpha^{h}$ channels of Ar_1415_ and Ar_149171_ subjected to an intense 2 fs pulse. In this study, the temporal profile of our pulses is assumed to be Gaussian and the pulse duration is given in FWHM. Both Ar_1415_ and Ar_149171_ are constructed as icosahedral structures.^48^ Kα and $K\alpha^{h}$ are x-ray emissions from 1+ and 2+ ions with single and double vacancies in K shell, respectively, and they are the two most dominant fluorescence channels found in our intense x-ray pulses.^48^ The energies of these lines obtained from the non-relativistic Hartree–Fock–Slater (HFS) model are 3050 and 3150 eV. As expected, Fig. 1 shows that the 2D fluorescence intensity distribution, ⟨I(k)⟩, of Ar_1415_ reveals a pattern with no structural information. On average, each excited Ar_1415_ cluster produces 0.0565 Kα and 0.0572 $K\alpha^{h}$ photons per atom per pulse over a 4*π* solid angle. In comparison, the Kα and $K\alpha^{h}$ yields found in an isolated Ar atom exposed to the same pulse are 0.048 and 0.040. The higher Kα and $K\alpha^{h}$ yields found in Ar_1415_ are the result that the recombination pathways, in addition to the direct photoionization pathway, are available in extended systems, as mentioned earlier.

The fact that Kα and $K\alpha^{h}$ have similar yields indicates that the x-ray interaction is not in the x-ray linear regime. In the linear regime (weak-field regime), the $K\alpha^{h}$ yield will be much lower than the Kα yield since the processes to reach the Kα and $K\alpha^{h}$ fluorescing states are one and two photon processes, respectively. Typically, XFEL pulses with fluence higher than the single ionization saturation fluence can induce nonlinear processes (i.e., sequential multiphoton absorption). In the nonlinear regime, the ionization dynamics is dominated by a sequential multiphoton process and the rate of double core-hole state (DCHS) production can be higher than the inner-shell decay rate of the single core-hole state (SCHS). In this case, significant depletion of the SCHS population can take place to produce DCHS during the pulse. As a result, relative to the linear regime, Kα has a lower yield and $K\alpha^{h}$ has a higher in the nonlinear regime.

**FIG. 2.**
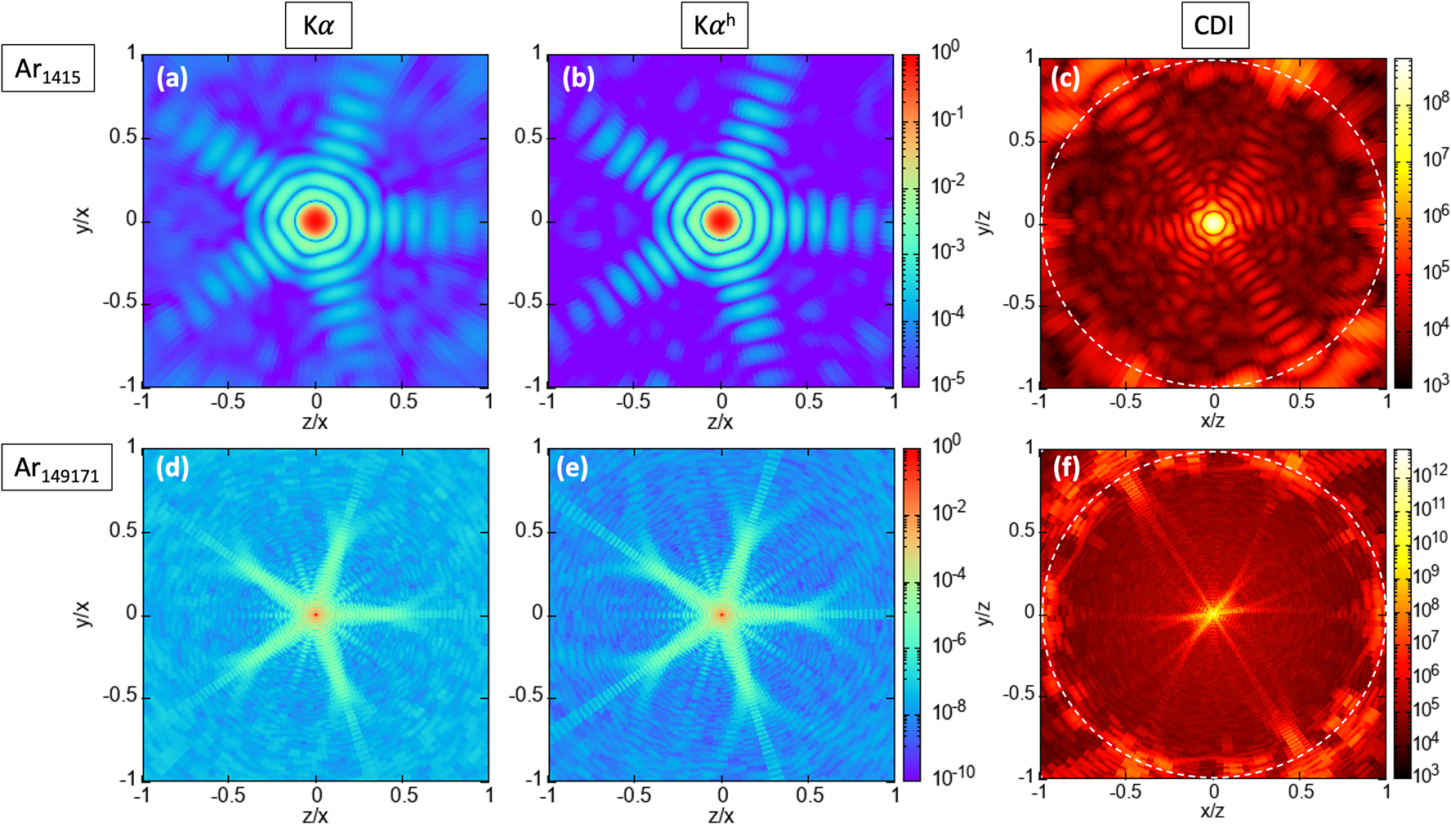
Fluorescence intensity correlation and scattering patterns of Ar clusters in an intense x-ray pulse. Panels (a)–(c) plot ${\left\langle G_{2}({\vec{k}}_{1},{\vec{k}}_{2},\{{\vec{R}}_{f,j}\})\right\rangle }_{\mathrm{avg}}$-1 of Kα, ${\left\langle G_{2}({\vec{k}}_{1},{\vec{k}}_{2},\{{\vec{R}}_{f,j}\})\right\rangle }_{\mathrm{avg}}$-1 of $K\alpha^{h}$ and the total differential scattering cross section of Ar_1415_ [Eq. (8)], respectively, exposed to an 2 fs, 5 keV, and 3.5 × 10^12^ photons/*μ*m^2^ pulse. Panels (d)–(f) in the bottom row are the same as those in the top row, except they are for Ar_149171_. The fluence corresponds 10 times the single-ionization saturation fluence of Ar. The geometry of the fluorescence and scattering detector is shown in Fig. 1. The FICs are computed with a fixed ${\vec{k}}_{1}$, which points along the −x axis, and ${\vec{k}}_{2}$ scans across all detector pixels on the y-z plane. In this geometry, the FIC images reveal the fivefold symmetry of the icosahedral structure of Ar_1415_ and Ar_149171_. The dashed circles (c) and (f) show the scattering angle of 45°. We note that the color scale of the total differential scattering cross section is given in units of classical electron radius squared, whereas the color scale of ${\left\langle G_{2}({\vec{k}}_{1},{\vec{k}}_{2},\{{\vec{R}}_{f,j}\})\right\rangle }_{\mathrm{avg}}$-1 is dimensionless.

Unlike the average intensity profile of fluorescence, which is I_0_, the computed FICs contain structural information as shown in Figs. 1 and 2. Despite having the same number of photons, the FIC from the $K\alpha^{h}$ channel has a higher contrast than that of Kα. This is because $K\alpha^{h}$ is emitted over a narrow time window in which the atomic motion is limited, whereas Kα spans a longer time emission window, which encompasses a larger atomic motion. For larger systems, $K\alpha^{h}$ is more advantageous for structural determination than Kα channel. For example, Ar_149171_, $K\alpha^{h}$ emission count is 1.5 times larger than that of Kα emission and the FIC of $K\alpha^{h}$ reveals higher contrast than that of Kα, as shown in Figs. 2(d) and 2(e).

**FIG. 3.**
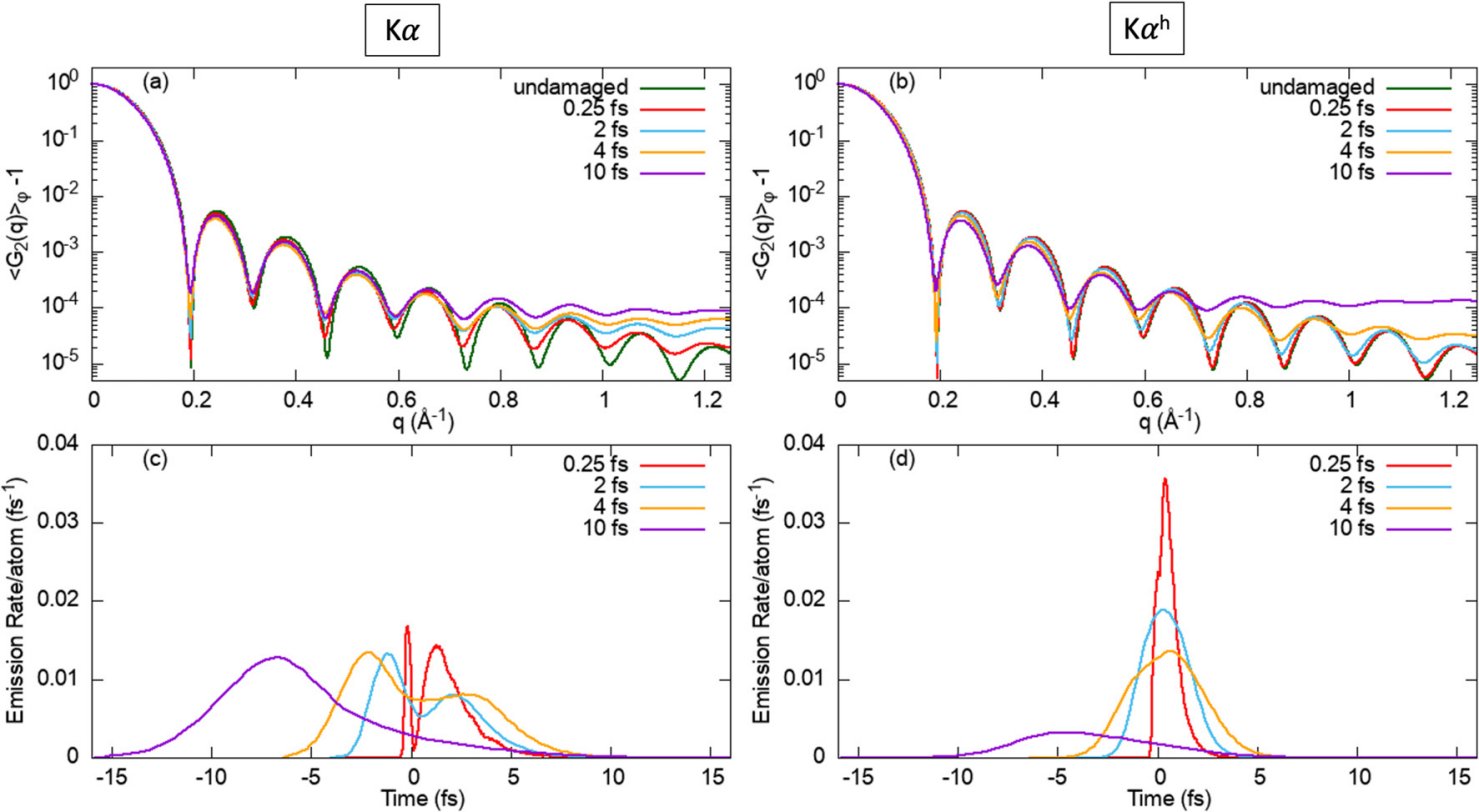
Pulse duration dependence of FIC computed from (a) Kα and (b) $K\alpha^{h}$ channels of Ar_1415_ exposed to 5 keV, 3.5 × 10^12^ photons/*μ*m^2^ pulse. For reference, the FICs computed from undamaged clusters are included. The bottom rows are the temporal emission profile of (c) Kα and (d) $K\alpha^{h}$ channels. See Table I for the total number of Kα and $K\alpha^{h}$ fluorescence photon/atom emitted per pulse.

Next, we investigate the pulse duration dependence of the FICs from Kα and $K\alpha^{h}$ of Ar_1415_. Figures 3(a) and 3(b) show the azimuthally averaged $G_{2}(q_{f})$ for Kα and $K\alpha^{h}$ emissions

$${\left\langle G_{2}(q_{f})\right\rangle }_{\varphi }=\frac{1}{2\pi }\int d\varphi {\left\langle G_{2}({\vec{k}}_{1},{\vec{k}}_{2},\{{\vec{R}}_{f,j}\})\right\rangle }_{\mathrm{avg}},$$ (14)

where $q_{f}=|{\vec{k}}_{1}-{\vec{k}}_{2}|$. For both of these emissions, we compute the FICs using 4 pulse durations of 0.25, 2, 4, and 10 fs. The 0.25 fs calculations were motivated by the recently available XFEL pulses.^50,51^ For comparison, we include the FICs calculated without damage (i.e., the nuclei are assumed to be frozen). The same pulse fluence of 3.5 × 10^12^ photons/*μ*m^2^ and photon energy of 5 keV were used in these calculations. The intensity of our pulse parameters is in the range of 10^19^–10^21^ W/cm^2^. We point out that required pulse intensities can be reduced by about a factor of 5 if a lower photon energy is used (close to the K-edge of SCHS of Ar) to achieve 10 times the single ionization saturation fluence. Figure 3(a) shows that the degree of deviation of the FIC of Kα from that of the undamaged case increases with pulse duration. Also, the degree of deviation increases with q, which is inversely proportional to the resolution in real space. Our results show that a shorter pulse in the few- and sub-femtosecond range will enable higher resolution and higher contrast imaging with the FIC from Kα. (See Appendix B for a detailed discussion about the correlation of cluster excitation and fluorescence dynamics.)

The examination of the pulse duration dependence of the FIC from $K\alpha^{h}$ emission, as shown in Fig. 3(b), reveals the same trend found in Kα emission. However, the FICs of $K\alpha^{h}$ are less sensitive to radiation damage. For example, the q-dependence FIC of $K\alpha^{h}$ at 0.25 fs is nearly identical to that of the undamaged case, whereas the q-dependence FIC of Kα at 0.25 fs deviates from the undamaged profile starting at q > 0.4 Å^−1^ already. Their difference is the result that the $K\alpha^{h}$ temporal emission profile is different from that of Kα. Figures 3(c) and 3(d) show that for our pulses with a pulse duration of 0.25, 2, and 4 fs, the resulting temporal profile of Kα is broader than that of $K\alpha^{h}$. These temporal profiles of Kα show a double-peak structure due to the presence of two pathways: the photoionization pathway and recombination pathway. The contribution of the photoionization and the recombination pathways peaks before and after t = 0, which is the peak of the XFEL pulses. On the other hand, these temporal profiles of $K\alpha^{h}$ remain a single-peak structure due to the fact that the contribution of the photoionization and recombination has significant temporal overlap.^48^

Figure 3 further shows that the fluorescence dynamics in 10 fs pulse is different from that in shorter pulse durations. In particular, the temporal profile of Kα is a single-peak distribution, indicating that the Kα production is predominantly via the photoionization pathway. The negligible role of the recombination pathway is due to substantial structural damage and expansion occurring before the peak of the pulse, leading to a reduced probability of electron–ion recombination. We point out that the cluster in the 10 fs pulse expands faster because it becomes more highly charged at longer pulse durations. This counterintuitive pulse duration effect is related to the effect of intensity-induced transparency,^52^ or frustrated absorption^53^ (see Appendix B for a more detailed discussion). Figure 3 reveals the trend that the contribution of the recombination pathway is increasingly diminished in a longer (less intense) pulse. Also, in a 10 fs pulse, the $K\alpha^{h}$ yield is about a factor 4 smaller than the Kα yield. The relatively small $K\alpha^{h}$ yield in the 10 fs pulse is because the DCHS production rate is smaller than the inner-shell decay rate of the SCHS. Thus, the 10 fs pulse will lead to a higher SCHS population but a smaller DCHS population. As a result, the $K\alpha^{h}$ yield is lower than the Kα yield in the 10 fs pulse. However, the situation is different for the shorter pulse durations, where the DCHS population rate is higher than the inner-shell decay rate. This leads to the result that the $K\alpha^{h}$ yield is higher than the Kα yield for 0.25 and 2 fs pulses.

Our analysis shows that there is a trade-off between high-resolution FIC images and the fluorescence intensity obtained with an intense pulse. As pointed out earlier, a shorter pulse (0.25 vs 2 fs pulse) produces higher resolution and spectral contrast FIC images, but it also leads to a lower fluorescence intensity per pulse, as shown in Table I. For experimental considerations, the FIC imaging with few-femtosecond pulses might be experimentally more efficient than with sub-femtosecond pulses since the latter tend to come with a lower number of photons.

**TABLE I.** The number of Kα and $K\alpha^{h}$ photons/atom produced over 4*π* solid angle in Ar_1415_ exposed to a 5 keV and 3.5 × 10^12^ photons/*μ*m^2^ pulse as a function of pulse duration. The numbers in the parentheses indicate the average number of fluorescence photons scattered into the forward scattering detector along z axis, as shown in Fig. 2(c).

Duration (fs)	Kα	$K\alpha^{h}$
0.25	0.0424 (10.0)	0.0431 (10.2)
2	0.0565 (13.3)	0.0572 (13.5)
4	0.0883 (20.8)	0.0622 (14.7)
10	0.0827 (19.5)	0.0212 (5.0)

**FIG. 4.**
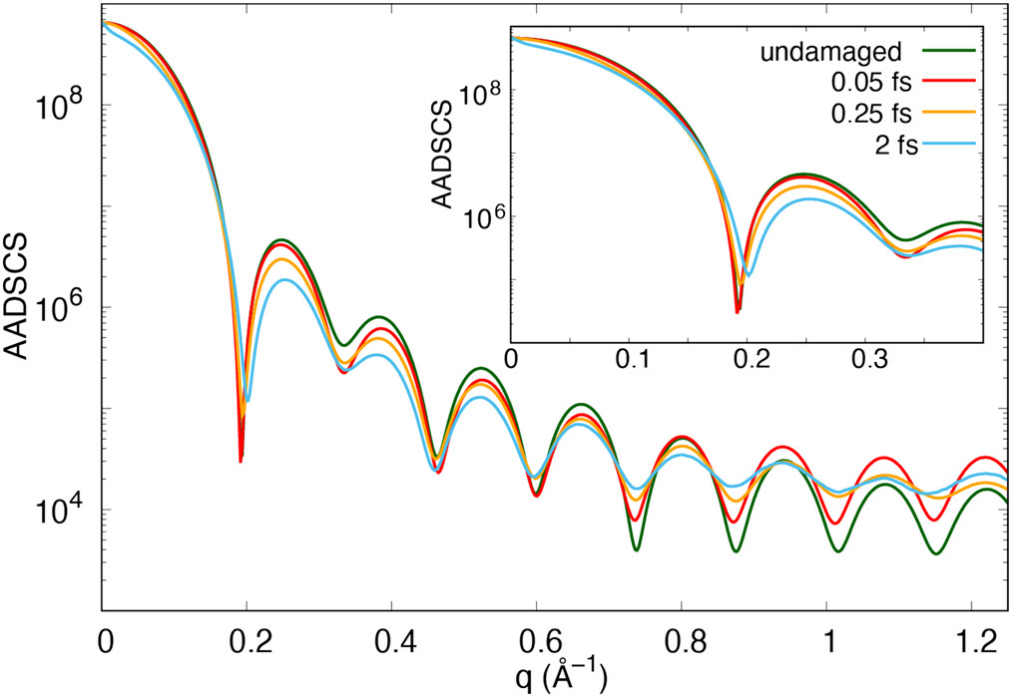
Pulse duration dependence of azimuthally averaged differential scattering cross section (AADSCS) of Ar_1415_ exposed to intense 5 keV x-ray pulses with a fluence of 3.5 × 10^12^ photons/*μ*m^2^. The inset shows that the AADSCS from a 2 fs pulse deviates from that of the undamaged sample already at a small q.

Unlike the FIC, the quality of the scattering pattern benefits substantially from sub-femtosecond pulses in comparison with that from femtosecond pulses. Figure 4 plots the pulse duration dependence of the azimuthally averaged differential scattering cross sections (AADSCS), $\frac{1}{2\pi }{\left\langle \frac{d\sigma_{\mathrm{total}}}{d\Omega }(q)\right\rangle }_{\varphi }$, in units of classical electron radius squared. The scattering pattern from a 2 fs pulse deviates substantially from that computed by assuming no electronic and structural damage. In particular, their difference starts immediately after the forward scattering region and the location of the first minimum shifts to a larger q. This shift is a result that ultrafast ionization leads to significant distortion in the electron distribution of Ar clusters and a large number of delocalized electrons. We note that the background in the scattering patterns in Figs. 2(c) and 2(f) is due to the scattering from these delocalized electrons with a small contribution from the incoherent scattering of atoms and ions.^11^ In contrast, using the same 2 fs pulse, the computed Kα and $K\alpha^{h}$ FICs, which include the effect of atomic motion over a time window larger than the XFEL pulse, deviate from the corresponding FIC of the undamaged cases only at high q. This means that the degree of atomic motion during a 2 fs pulse is not significant, and this further suggests that the expanding delocalized electron cloud is the main factor that reduces the quality of the scattering image in a 2 fs pulse. To further improve the quality of the scattering images of the Ar cluster, one can employ sub-femtosecond pulses. As shown in Fig. 4, the degree of radiation damage, that is, the extent of the expansion of the electron clouds and the nuclear motion, in 0.25 and 0.05 fs pulses is small. With reduced damage, the scattering efficiency per pulse and scattering cross section will also increase.

Practically, our calculations show that about 60 photons are scattered into the detector from Ar_1415_ exposed to a 5 keV, 2 fs, 3.5 × 10^12^ photons/*μ*m^2^ pulse. In each XFEL pulse, about 50 of these photons are scattered in the forward direction and in a small q region (q < 0.2 Å^−1^) and only 10 photons are found beyond the forward scattered peak. In comparison, about 13 Kα and 13.2 $K\alpha^{h}$ photons are emitted into the scattering detector region (not including emission from other fluorescence channels). This suggests that, already in the small q region (i.e., beyond the first peak), the scattering signals and fringes will be strongly contaminated by the fluorescence photons. From the perspective of imaging small particles (molecules), fluorescence can be a dominant noise in the scattering pattern. One can in principle increase the scattering intensity with a higher fluence pulse, but there is a trade-off between the higher scattering signal and the quality of the scattering pattern. The quality of the scattering pattern degrades at higher fluence because of the presence of more delocalized electrons and their fast expansion. We note that the achievable focal intensities are usually experimentally limited. These calculations on small Ar clusters show that they will be challenging to image with the CDI approach because the fluorescence signal is comparable or larger. For larger systems, the impact of fluorescence on scattering images will be less severe and it will be limited to a large q region as the scattering signal (total cross section) scales with $N_{a}^{4/3}$ (as discussed in work by Kirz and co-workers^54^), whereas the fluorescence signal scales with *N_emitter_*.

### B. Doped iron oxide nanoparticle

**FIG. 5.**
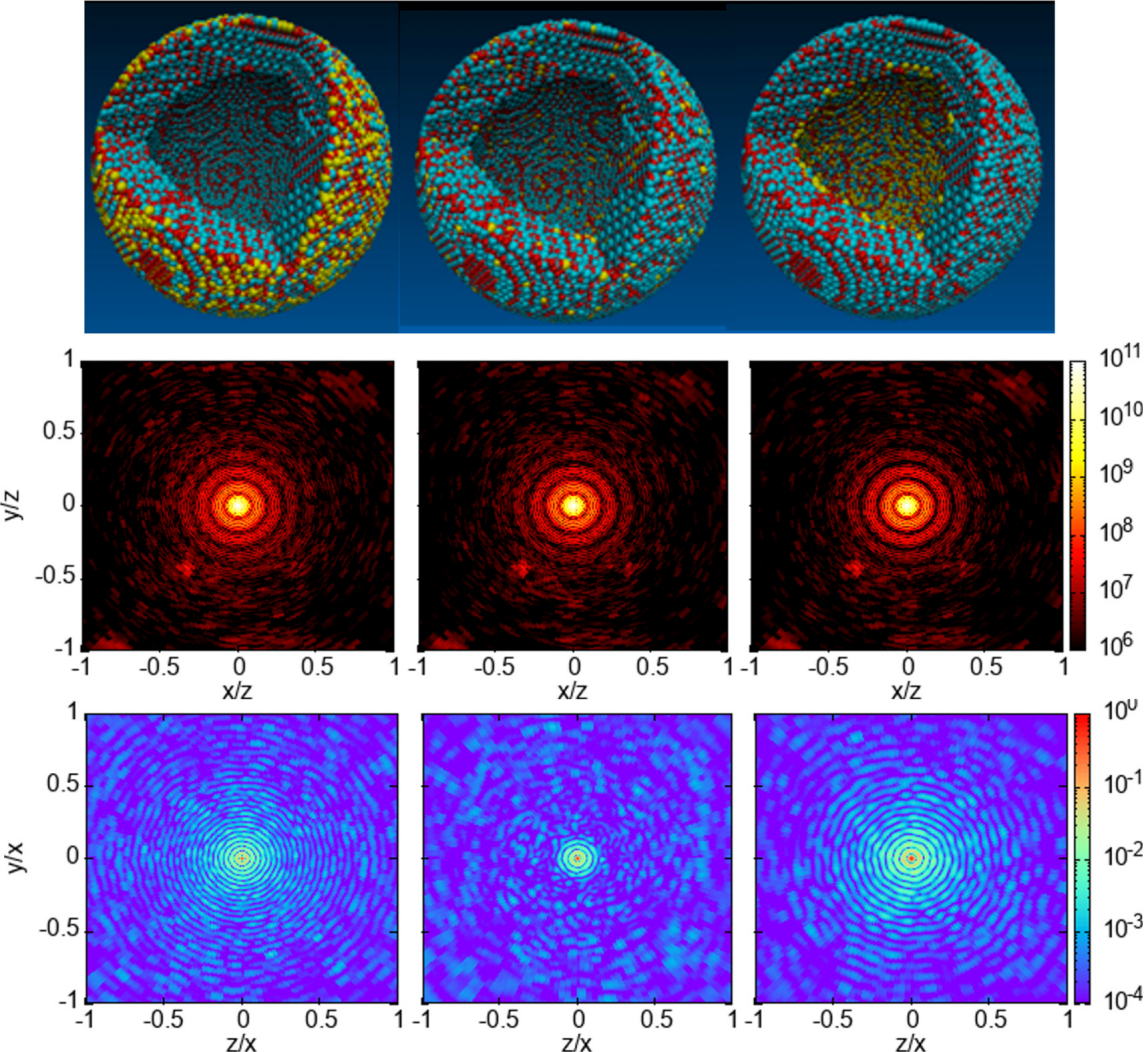
Top panel: Structures of hollow core–shell γ−Fe_2_O_3_ nanoparticles with Mo-dopants distributed randomly in the outer layer (left) and everywhere (middle) and inner layer (right) in the nanoparticle. The yellow, cyan, and red dots are molybdenum, iron, and oxygen atoms. The panels in the second row are the corresponding differential scattering cross sections computed from an intense 2 fs, 5 keV XFEL, 6 × 10^11^ photons/*μ*m^2^ pulse in units of classical electron radius squared. The fluence is 5 times the single-ionization saturation fluence of the Mo atom, but it is below the saturation fluence of O and Fe. The spot near (−0.4, −0.5) in each pattern is a Bragg peak, which is sensitive to the orientation of the cluster with respect to the x-ray propagation. The panels in the third row show the FICs given by G_2_-1 computed from Lα fluorescence channel of the Mo atom. The same pulse parameter set is used for the FICs. The scattering detector is perpendicular to the fluorescence detector, and their geometry is shown in Fig. 1.

Next, we explore CDI and FIC for imaging of elemental contrast in heterogeneous samples. Our hollow core–shell structure of *γ*-Fe_2_O_3_ has a polycrystalline structure with an inner radius and outer radius of 6 and 8.5 nm. We considered three different dopant distributions, as shown in Fig. 5. In the first structure, the Mo dopants are located on the outermost 0.2 nm (i.e., between the radii of 8.3 and 8.5 nm) of the nanoparticle. In the second structure, the dopants are randomly distributed in the sample volume. In the third structure, the Mo atoms are doped in the inner 0.4 nm layer (i.e., between the radii of 6.0 and 6.4 nm). In all these three nanoparticles, the relative abundance of the Mo atoms (the ratio of the number of the Mo atoms and the sum of the Mo and Fe atoms) is 6%.

Each of these NPs has about 65 100 iron, 92 300 oxygen, and 4100 molybdenum atoms. Together with the electrons, each MC/MD calculation tracks more than 2.7 × 10^6^ particles. These calculations were performed on the high-performance computer, Mira, at ALCF. We calculate the scattering pattern and fluorescence intensity correlation patterns using a 2 fs, 5 keV pulse. The pulse fluence is chosen to be 5 times the saturation fluence of the single ionization of the Mo atom. At this fluence level of 6 × 10^11^ photons/*μ*m^2^ and photon energy, the probability of photoionization for iron and oxygen atoms is below saturation. We note that our pulse reaches an intensity of 2.6 × 10^19^ W/cm^2^. The photon energy is chosen such that it is far from the resonances of the ground state and excited state hidden resonances in Fe, Mo, and O, and it allows us to study the L-shell fluorescence emission of Mo. We note that our XFEL parameters in the non-resonant regime are not optimal for imaging elemental contrast with CDI. Anomalous diffraction can be explored to study the scattering response of our Mo-doped NPs.

Our calculation shows that there are only small differences among the scattering patterns of these three structures, indicating that the scattering signals are not very sensitive to the dopant distribution in Fig. 5. These small differences are a result of the scattering signals being dominated by Fe and O atoms, in which their distributions are similar in these hollow-core NPs. We note that the scattering amplitude can be considered as the superposition of two out-of-phase scattering amplitudes from two spherical particles with radii of 8.5 and 6 nm. This superposition gives a beat pattern in Fig. 5(a).

**FIG. 6.**
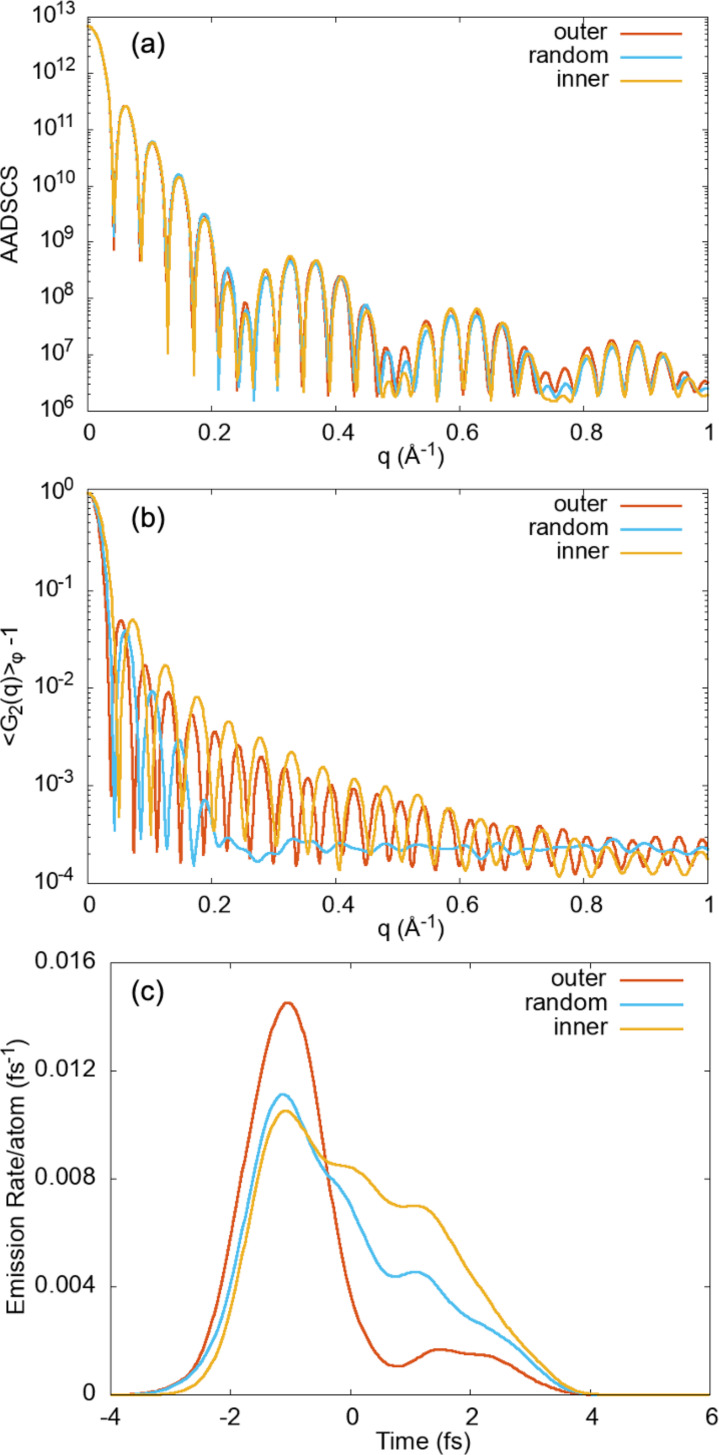
(a) Azimuthally averaged differential scattering cross section (AADSCS) and (b) azimuthally averaged G_2_(q)-1 as a function of momentum transfer, q, of the three iron oxide NPs with different dopant distributions shown in Fig. 5. (c) Temporal emission profiles of Lα emission. The same pulse parameters of 2 fs, 5 keV XFEL, and 6 × 10^11^ photons/*μ*m^2^ were used.

Unlike the scattering processes, the fluorescence emission is element specific and electronic transition specific in the non-resonant regime. Figures 5(c) and 6(b) show that the FICs computed from the Lα channel of Mo reveal distinct fingerprints for these NPs with different dopant distributions already in the small q region. The energy obtained from the non-relativistic HFS model for Mo Lα is 2350 eV. Here, the pulse parameter is the same pulse parameter used in the scattering calculations. In comparison, the average number of Lα photons produced per pulse from each Mo atom is about 0.0351, 0.0332, and 0.0278 for inner-doped, randomly doped, and outer-doped structures. These correspond to 140, 136, and 114 photons over 4*π* solid angle. These different yields are the result of the temporal emission profile and the relative contribution of the two Lα pathways (direct photoionization and recombination pathways) depending on the location of the Mo atoms within the NP. In general, for the Mo atoms residing near the surface layers of the NP, the Lα events are mostly via the direct photoionization pathway and they take place earlier in the pulse [before t = 0, as shown in Fig. 6(c)]. However, the Mo atoms residing deeper in the NP have a higher chance of undergoing Lα via the recombination pathway in addition to the photoionization pathway and thus have a broader temporal emission profile.

Based on their photoabsorption cross section (10 and 0.37 kb for Fe and O^55^) at 5 keV, and the fluorescence yield (0.006 and 0.007 for Fe and O^55^), the number of O-atom Kα photons (about 525 eV) is found to be about 15 per NP per pulse over the 4*π* solid angle or 2.5 photons into the fluorescence detector, whereas the number of Fe-atom L-shell emission photons (about 705 eV) is found to be about 390 per NP per pulse or 65 photons into the fluorescence detector. With an energy resolving pixelated detector, one can distinguish these fluorescence events from those originating from the Mo atoms (about 2350 eV). Without such a detector, the Fe fluorescence can contribute significant noise to the FIC obtained with Lα of Mo. We also point out that using the lower energy threshold on a photon-counting detector, one should be able to distinguish the Mo vs the Fe and O fluorescence due to their large energy separation. Also, one can potentially suppress Fe fluorescence by using a lower fluence. For example, by reducing the fluence by a factor of 5 (i.e., the single ionization saturation of Mo), the number of Fe fluorescence will reduce by a factor of 5 to about 78 photons over 4*π*, whereas the number of Lα of Mo is estimated to be about 150 photons based on the Mo L-shell fluorescence yield of 0.04.^55^

## IV. SUMMARY

In summary, we investigated FIC and CDI for imaging high-resolution structure of nanosized, non-periodic particles. Using Ar clusters as prototypical systems, we investigated the pulse duration dependence of the FIC approach from Kα and $K\alpha^{h}$ channels. We showed that the temporal emission profiles and the number of fluorescence photons depend strongly on the XFEL pulse duration, suggesting that the pulse duration can serve as a control knob to optimize the fluorescence dynamics for the FIC imaging. Also, we showed that, in few-femtosecond or sub-femtosecond pulses, the resulting FICs from $K\alpha^{h}$, in general, reveal a higher contrast than those from Kα due to $K\alpha^{h}$ having narrower temporal emission profiles. In comparison with the result from the 2 fs pulse, the FIC from a 0.25-fs pulse offers only a slightly higher degree of contrast, but the total fluorescence intensity count is lowered by a factor of 2, suggesting that few-femtosecond pulses may be sufficient for the FIC measurements. On the other hand, it is more advantageous to perform scattering experiments with an attosecond pulse than with a 2 fs pulse as the short pulse reduces the sample electronic and structural damage, which leads to a higher scattering cross section and higher quality of scattering signals. Due to the isotropic nature of fluorescence emission, the number of fluorescence photons reaching the scattering detector can be larger than the number of scattered photons beyond the first peak. Thus, fluorescence can be a dominant noise in the scattering pattern at the high q region.

More interestingly, we presented the FIC of heterogeneous nanoparticle γ−Fe_2_O_3_ structures with three different Mo dopant distributions in an intense x-ray pulse. We showed that, while the scattering signals in the non-resonant regime are dominated by the Fe and O atoms and possess only small differences between the three structures, the FICs from Lα of Mo illuminate the distribution of the Mo dopant. Our work suggested that the FIC can be exploited for imaging the distribution of trace elements in non-resonant regime. In the future, K-shell excitation in Mo, which requires 20 keV photons, will also be considered since XFEL pulses with photon energies up to 25 keV will be accessible at the EuXFEL.

We point out that the current FIC analysis made use of a subset of 2-point correlation functions. For future work, it will be useful to exploit the full volumetric data from a fixed sample orientation for structure analysis and determine the number of sample orientations needed for 3D structural reconstruction. Also, it will be interesting to explore higher-order coherence, G^(*N*)^ ( ${\vec{k}}_{1}$, …, ${\vec{k}}_{N}$),^17,23^ derived from the x-ray fluorescence of a collection of atoms exposed to intense XFEL pulses.

## Figures and Tables

**FIG. 7.**
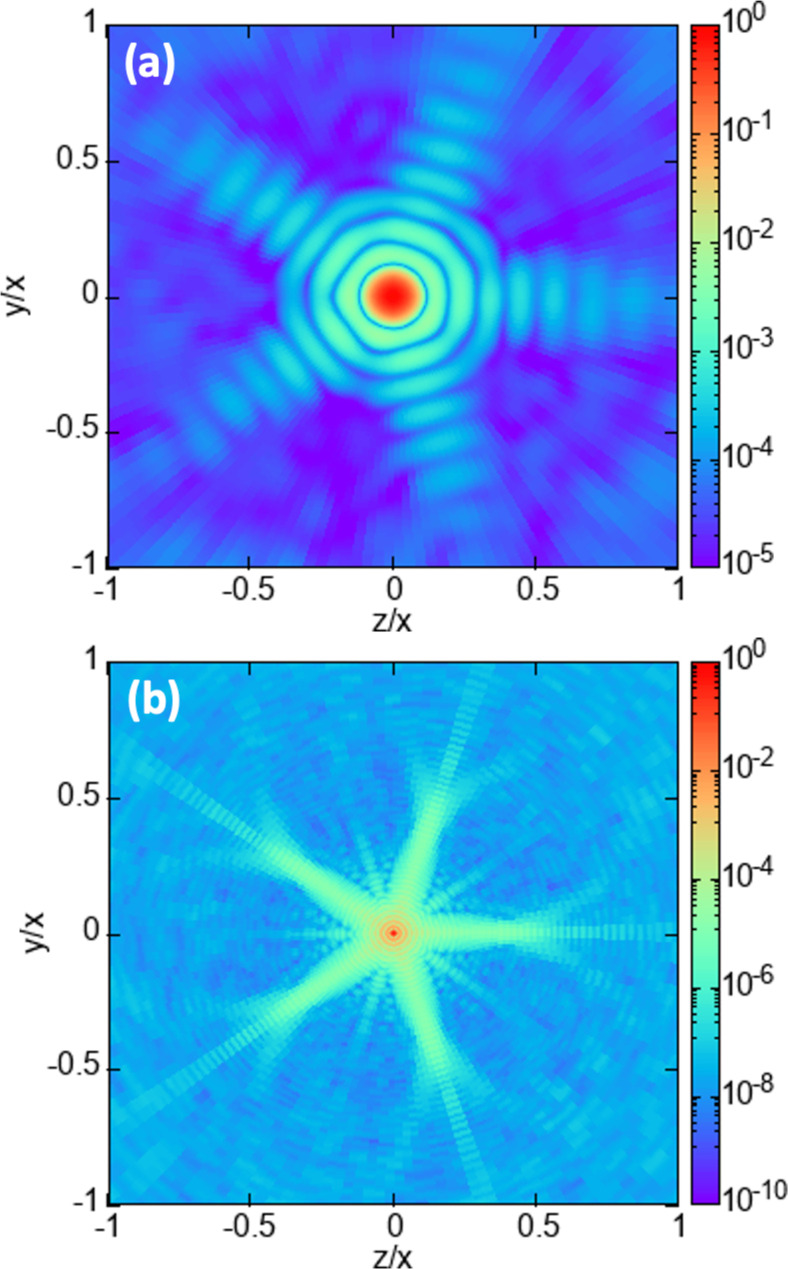
FIC ( ${\left\langle G_{2}({\vec{k}}_{1},{\vec{k}}_{2},\{{\vec{R}}_{f,j}\})\right\rangle }_{\mathrm{avg}}$-1) of the sum of Kα and $K\alpha^{h}$ signals for (a) Ar_1415_ and (b) Ar_149171_. The pulse parameters are same as those in used in Fig. 2.

**FIG. 8.**
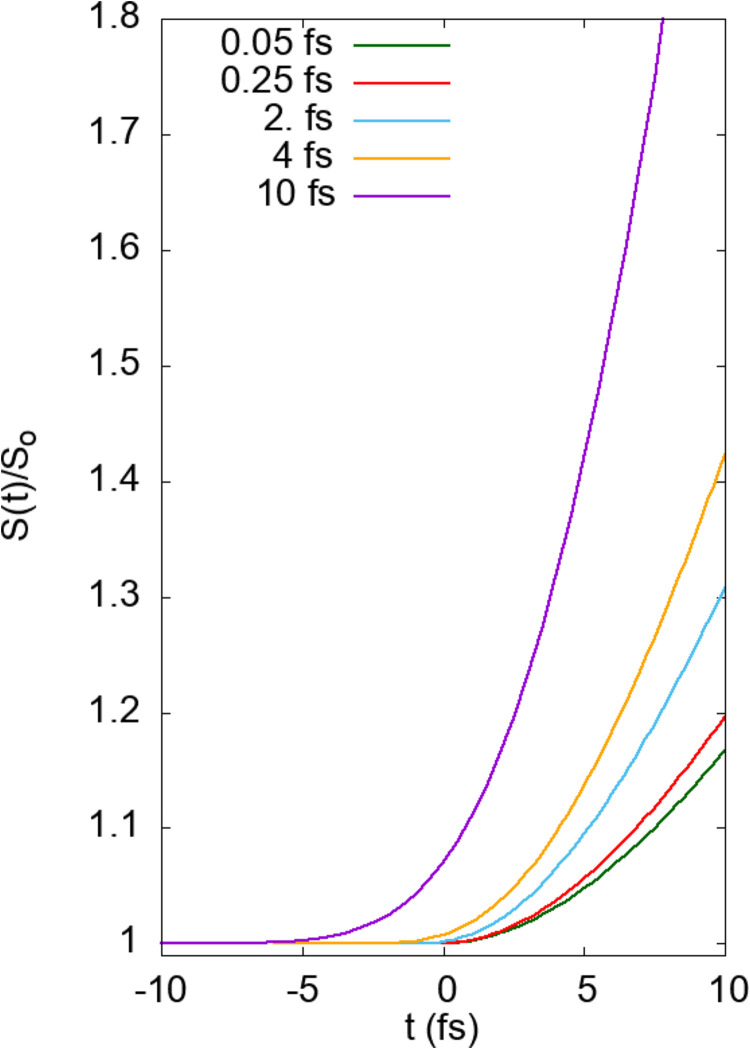
Expansion of the cluster. Ratio of the rms radius, S(t), to the undistorted structure, S_0_, as a function of pulse duration. For all these pulses, the photon energy is 5 keV and the fluence 3.5 × 10^12^ photons/*μ*m^2^.

## Data Availability

The data that support the findings of this study are available from the corresponding author upon reasonable request.

## APPENDIX A: SUM FIC OF Kα AND $K\alpha^{h}$

Since there are currently no energy-resolved pixelated detectors in common use for CDI experiments, Fig. 7 shows the FIC signals that include the contribution of both Kα and $K\alpha^{h}$ channels for Ar_1415_ and (b) Ar_149171_. These plots show that the contrast is reduced in comparison to those shown in Fig. 2.

## APPENDIX B: PULSE DURATION DEPENDENCE OF IONIZATION/EXPANSION, FLUORESCENCE, AND SCATTERING DYNAMICS OF Ar CLUSTER

In this paper, we investigate the FIC and CDI as a function of pulse duration. Using MC/MD method, we follow the cluster dynamics during and after the pulse. To show the structural dynamics of Ar_1415_, Fig. 8 plots the ratio of the rms radius, S(t), of the XFEL-excited cluster and of that of the undistorted structure, *S*_0,_ as a function of pulse duration. For all these pulses, the photon energy is 5 keV and the fluence is 3.5 × 10^12^ photons/*μ*m^2^. In these pulses, the cluster undergoes structural distortion in femtoseconds, but the details of the dynamics depend on the pulse duration.

For our Ar_1415_ cluster, Fig. 8 shows that in a 10, 4, 2, 0.25, and 0.05 fs pulse, its size has increased by 10% at t = 0.5, 4, 5, 7, and 7.5 fs, respectively, where t = 0 is the peak of the pulse. This suggests that substantial distortion takes place during the pulse with 4 and 10 fs pulse duration. On the other hand, for the shorter pulses, the structure of the cluster remains largely intact during the pulse and a significant structural distortion takes place only after the pulse.

It is clear from Fig. 8 that the cluster expands faster in a longer pulse. This is because it is charged to a higher charge state in a longer pulse. We note that the sequential multiple x-ray ionization of atoms in intense pulses is pulse duration-dependent—shorter (higher intensity) pulses can suppress ionization. This is known as intensity-induced transparency^52^ or frustrated absorption.^53^ This happens when photoabsorption cross section is reduced during the pulse due to the loss of core–shell electrons. For argon, the lifetime of the single and double core-hole state is of the order of 1 fs. Thus, in a 10 fs pulse, the inner decay, mostly Auger decay (A), can take place to refill the core hole created by the initial photoionization (P). The resulting ions can then undergo further photoionization. The sequence of PAPA… events can efficiently charge the atoms to a very high charge state, in addition to electron-impact ionization. In comparison, in a 0.25 fs pulse, the degree of x-ray ionization is smaller as the photoabsorption cross section of the transiently created ions is smaller. As a result, the cluster expands faster in a longer pulse duration.

Next, we discuss the effects of pulse duration on the CDI and the FIC method. In general, CDI “sees” the nuclear structure (bound electron) and distribution of delocalized electrons during the pulse. The motion of the delocalized electron would produce background in the scattering patterns and reduce the contrast of the scattering fringes. One would expect that a shorter exposure (pulse duration) would capture the instantaneous/frozen target structure and the obtained scattering pattern would better reflect the undamaged structure with a higher contrast. Our result that shows a shorter pulse will enable higher resolution imaging with higher contrast. This result is not too surprising as it falls within the expectation of a simple view that a short exposure allows the photons to “see” a frozen structure. Our results, which obtained from the MC/MD model that accounts for the full electronic and nuclear dynamics, show that this view holds even in very intense pulses. Yet, to “freeze” the expansion of the delocalized electron cloud, as well as the structure, during the pulse, few-femtosecond pulses are needed.

On the other hand, the FIC is not sensitive to the distribution of the delocalized electrons, but it is sensitive to the population of the fluorescence state. For Kα and $K\alpha^{h}$, their fluorescence states are singly and doubly charged ions with one and two K-shell vacancies, respectively. These Kα and $K\alpha^{h}$ fluorescence states have lifetimes of about 1 and 0.5 fs, respectively. For a 2 fs and longer pulse, which are longer than these core-hole state lifetimes, Auger decay/fluorescence can take place during the pulse. On the other hand, one would expect that with pulses shorter than the core-hole lifetime, the probability of Kα will be reduced as the population of Kα state will be depleted through further ionization events. As an example, 0.25 fs pulse has 8 times higher ionization rate than 2 fs, based on isolated atomic model, one might simply expect that the Kα yield in 0.25 fs would be 8 times smaller than that from a 2 fs pulse. However, we found that the Kα is only reduced by a factor of 0.82. This is because fluorescence dynamics in extended systems are different compared to those in an isolated atom; fluorescence pathways via electron–ion recombination are available, in addition to the photoionization pathways, which are found in isolated atoms.

For ions that emit Kα via the recombination pathways in a 2 fs, 5 keV pulse, they are charged to an average charge state of about 4+ rapidly during the pulse.^48^ One example recombination pathway is that an Ar atom is first ionized to reach an electronic configuration of 1s 2s^2^ 2p^3^ 3s^2^ 3p^6^ before undergoing 3 recombination events to reach Kα fluorescence state (i.e., SCHS). Figure 3(c) shows the temporal emission profiles of the Kα emission. The peaks before and after t = 0, which is the peak of the XFEL pulses, are due to the Kα via photoionization and recombination pathways, respectively. Careful examination of this figure shows that the contribution of photoionization pathways decreases almost linearly with the pulse duration, similar to the prediction of the isolated atomic model. On the other hand, the contribution of the recombination pathways decreases with increasing pulse duration. The recombination pathways depend on the cluster dynamics. In a 10 fs pulse, the role of the recombination pathway is small because substantial structural damage and expansion occurs before the peak of the pulse, leading to a reduced probability of electron–ion recombination.

The dynamics of $K\alpha^{h}$ is more complicated than Kα. Despite showing a single-peak distribution in a 0.25, 2, and 4 fs pulse, both the photoionization and the recombination pathways contribute to the $K\alpha^{h}$ production, as shown in our previous work.^48^ This is because the two pathways have significant temporal overlap. It is not that the recombination rate is faster in $K\alpha^{h}$. Rather the number of the recombination events needed to reach the $K\alpha^{h}$ fluorescence state (doubly charged ion) is typically one less than that needed to reach Kα fluorescence state (singly charged ion). One example of $K\alpha^{h}$ recombination pathway in a 2 fs, 5 keV pulse is that an Ar atom is first ionized to reach an electronic configuration of 2s^2^ 2p^4^ 3s^2^ 3p^6^ before undergoing 2 recombination events to reach $K\alpha^{h}$ fluorescence state (i.e., DCHS). We point out that ions that undergo $K\alpha^{h}$ recombination are also charged to an average charge state of about 4+, similar to that found in the recombination pathways of Kα.^48^ This means the $K\alpha^{h}$ recombination pathways take place earlier than those of the Kα recombination pathways. On the other hand, in comparison with Kα, the photoionization pathways of $K\alpha^{h}$ tend to take place later than those of Kα. This is because the photoionization pathways of $K\alpha^{h}$ require two-photon absorption events, whereas those of Kα require one-photon absorption event. As a result of the relative timing of the photoionization and recombination pathways, Kα and $K\alpha^{h}$ temporal emission profiles show a single- and double-peak distributions. Both Kα and $K\alpha^{h}$ temporal profiles are sensitive to the pulse parameters. For example, the $K\alpha^{h}$ profile can turn into a double-peak distribution with a higher fluence pulse.^48^

If we compare the Kα and $K\alpha^{h}$, all the pulse durations show comparable Kα and $K\alpha^{h}$ yields, except in a 10 fs pulse, where the $K\alpha^{h}$ yield is a factor of 4 lower. The relatively small $K\alpha^{h}$ yield in the 10 fs pulse is because the DCHS production rate is smaller than the inner-shell decay rate of the SCHS. Using the HFS model, the inner-shell decay rate (Auger and fluorescence) of the single core-hole state is found to 0.025 a.u., about 2.0 times higher than the K-shell photoionization rate that produces the double core-hole state in the 10 fs pulse.

As a result, the $K\alpha^{h}$ yield is lower than the Kα yield in the 10 fs pulse. However, the situation is different at shorter pulse durations, where the DCHS population rate is higher than the inner-shell decay rate. This leads to the result that the $K\alpha^{h}$ yield is higher than the Kα yield in the 0.25 and 2 fs pulse.
